# Sympathetic and hemodynamic responses to exercise in heart failure with preserved ejection fraction

**DOI:** 10.3389/fcvm.2023.1148324

**Published:** 2023-04-17

**Authors:** Kazumasa Manabe, Andrew W. D’Souza, Takuro Washio, Ryosuke Takeda, Sarah L. Hissen, John D. Akins, Qi Fu

**Affiliations:** ^1^ Women’s Heart Health Laboratory, Institute for Exercise and Environmental Medicine at Texas Health Presbyterian Hospital, Dallas, TX, United States; ^2^ Cardiology Division, Internal Medicine, The University of Texas Southwestern Medical Center, Dallas, TX, United States; ^3^ Neurovascular Research Laboratory, School of Kinesiology, Western University, London, ON, Canada

**Keywords:** neural control of circulation, exercise intolerance, autonomic dysfunction, pressor response, sympathoexcitation, sympatholysis, low muscle blood flow

## Abstract

Excessive sympathetic activity during exercise causes heightened peripheral vasoconstriction, which can reduce oxygen delivery to active muscles, resulting in exercise intolerance. Although both patients suffering from heart failure with preserved and reduced ejection fraction (HFpEF and HFrEF, respectively) exhibit reduced exercise capacity, accumulating evidence suggests that the underlying pathophysiology may be different between these two conditions. Unlike HFrEF, which is characterized by cardiac dysfunction with lower peak oxygen uptake, exercise intolerance in HFpEF appears to be predominantly attributed to peripheral limitations involving inadequate vasoconstriction rather than cardiac limitations. However, the relationship between systemic hemodynamics and the sympathetic neural response during exercise in HFpEF is less clear. This mini review summarizes the current knowledge on the sympathetic (i.e., muscle sympathetic nerve activity, plasma norepinephrine concentration) and hemodynamic (i.e., blood pressure, limb blood flow) responses to dynamic and static exercise in HFpEF compared to HFrEF, as well as non-HF controls. We also discuss the potential of a relationship between sympathetic over-activation and vasoconstriction leading to exercise intolerance in HFpEF. The limited body of literature indicates that higher peripheral vascular resistance, perhaps secondary to excessive sympathetically mediated vasoconstrictor discharge compared to non-HF and HFrEF, drives exercise in HFpEF. Excessive vasoconstriction also may primarily account for over elevations in blood pressure and concomitant limitations in skeletal muscle blood flow during *dynamic* exercise, resulting in exercise intolerance. Conversely, during *static* exercise, HFpEF exhibit relatively normal sympathetic neural reactivity compared to non-HF, suggesting that other mechanisms beyond sympathetic vasoconstriction dictate exercise intolerance in HFpEF.

## Introduction

During exercise, the sympathetic nervous system plays a pivotal role in the maintenance of blood pressure (BP) and distribution of blood flow towards active skeletal muscle. Specifically, exercise-induced increases in sympathetic vasoconstrictor discharge promotes venous return to the heart which supports increases in left ventricular (LV) cardiac output ($\dot{Q}$_c_), and vasoconstriction in non-active tissues serves to redistribute blood flow toward active skeletal muscle to meet the metabolic demands of exercise (1). Conversely, locally released metabolic by-products within active skeletal muscle offset sympathetically-mediated vasoconstriction (i.e., functional sympatholysis) to ensure blood supply matches the metabolic requirements of active muscle (2, 3). This phenomenon is particularly important in the resistance arterioles of the muscle microcirculation (4). However, sympathetic nervous system hyper-reactivity and impaired peripheral vascular responses to exercise create an imbalance between vasoconstriction in non-active muscles and vasodilation in active muscles, ultimately resulting in a mismatch between oxygen supply and demand (5–8). Indeed, aberrant sympathetic neural reactivity and peripheral vascular responses to exercise are directly related to exercise intolerance and a reduced peak aerobic capacity or peak oxygen uptake ($\dot{V}$O_2peak_) in certain populations characterized by chronic disease (5–8).

Exercise intolerance is a hallmark of heart failure (HF) with preserved ejection fraction (LVEF ≥ 50%: HFpEF), and is directly related to morbidity, hospitalization, and mortality rates (9). Despite, HFpEF representing ∼50% of all HF cases worldwide (10), most studies have focused on the mechanisms underpinning exercise intolerance in patients with HF with reduced ejection fraction (LVEF ≤ 40%: HFrEF). Thus, the determinants of exercise intolerance in patients with HFpEF remain poorly understood. As a result, pharmacological therapies targeted toward improving exercise capacity are increasingly utilized in patients with HFrEF; however, few evidence-based therapies currently exist for patients with HFpEF (11, 12).

Emerging evidence indicates that the underlying mechanisms of exercise intolerance differ between patients with HFrEF and HFpEF, despite both forms of HF having a lower $\dot{V}$O_2peak_ than age-matched controls (5, 7, 13–15). Borlaug et al. (15, 16) found that $\dot{V}$O_2peak_ in patients with HFpEF was negatively correlated with peripheral vascular reserve (e.g., systemic vascular resistance, and microvascular function) and positively correlated to cardiac function [e.g., lower peak heart rate (HR), and reduced myocardial contractility]. Bhella et al. (13) observed no significant difference in cardiac function at peak exercise between patients with HFpEF and age-matched controls, as assessed by cardiac power output ($\dot{Q}$_c_ × mean BP) and stroke work (stroke volume (SV) × mean BP), which are further indicators of overall cardiac function and major determinants of exercise capacity (17–19), despite patients with HFpEF exhibiting a lower $\dot{V}$O_2peak_. These results indicate that unlike in patients with HFrEF who exhibit cardiac limitations, peripheral, but not central (cardiac), factors may primarily explain the lower $\dot{V}$O_2peak_ in patients with HFpEF (7, 20). A potential mechanism underpinning the greater vasoconstrictive response to exercise in patients with HFpEF, might be sympathetic nervous system hyperreactivity, which would lead to attenuate muscle blood flow *via* excessive vasoconstriction, thus impairing oxygen delivery to the contracting skeletal muscles, ultimately lowering $\dot{V}$O_2peak_.

Assessments of autonomic and vascular function during exercise are typically conducted using either dynamic exercise such as cycling or running, or static (i.e., isometric) exercise such as sustained handgrip. Notably, the physiological responses to these types of exercise are distinct (1, 21). The autonomic mechanisms contributing to the maintenance of BP during exercise, called pressor response (e.g., central command, skeletal muscle metaboreflex, arterial baroreflex, chemoreflex, hormonal response), are engaged in both dynamic and static exercise. However, the pressor response during contractions not only depends on muscle mass but the types of contraction (21): Dynamic exercise involves larger central (cardiac) and peripheral (limb vascular) hemodynamic responses, whereas static exercise better isolates peripheral hemodynamic limitations to exercise. To date, there is a paucity of information summarizing the sympathetic and hemodynamic responses to exercise including types of muscle contraction in patients with HFpEF, and how these neuro-cardiovascular responses relate to exercise intolerance in this patient population.

The current mini review focuses on the sympathetic neural and hemodynamic responses during dynamic and static exercise in patients with HFpEF compared to patients with HFrEF and non-HF controls. Further, we explored the relationship between sympathetic nerve activity and vasoconstriction in patients with HFpEF to advance our understanding of the mechanisms underpinning exercise intolerance in this group of individuals (see Table 1 for a summary of the references).

**Table 1:** Sympathetic and hemodynamic responses to dynamic and static exercise in HFpEF vs. HFrEF or non-HF.

Author, year	Population (female and male)/characteristics	Exercise type	Method of assessment	Major findings
**Sympathetic responses**				
***MSNA***				
Badrov et al., 2022 (22)	HFpEF = 1(F): 74 years, LVEF 63% CTRL = 1(F): 74 years, no history of CVD	4 min of dynamic 1-leg cycling (2 min each at mild and moderate intensity)	ΔMSNA BF from baseline with microneurography	HFpEF > CTRL during both mild and moderate intensity exercise
Bunswat et al., 2021 (23)	HFpEF = 1(F): 65 years, LVEF 70% CTRL = 1(F): 70 years, no history of CVD	2 min static handgrip at 30% and 40% MVC	ΔMSNA BF from baseline with microneurography	HFpEF = CTRL at the last 1 min of both handgrip bouts
***Plasma NE***				
Borlaug et al., 2006 (15)	HFpEF = 17 (16F1M): 65 ± 9 years, LVEF ≥ 50%, $\dot{V}$O_2peak_ 9.0 ± 3.4 ml/kg/min CTRL = 19 (16F3M): 65 ± 9 years, $\dot{V}$O_2peak_ 14.4 ± 3.4 ml/kg/min	Upright cycling until exhaustion	Plasma NE immediately after peak exercise	HFpEF = CTRL at peak exercise
Tsuchida et al., 2007 (24)	Preserved EF = 8 (3F5M): 57 ± 7 years, LVEF 67 ± 6%, $\dot{V}$O_2peak_ 25.7 ± 6.9 ml/kg/min Reduced EF = 12 (2F10M): 46 ± 13 years, LVEF 33 ± 10%, $\dot{V}$O_2peak_ 18.2 ± 2.0 ml/kg/min	Sitting cycling until exhaustion	Plasma NE during exercise	Preserved EF = Reduced EF at peak exercise
**Hemodynamic responses**				
***Blood pressure***				
Kato et al., 2008 (8)	HFpEF = 20 (12F8M): 70 ± 5 years, LVEF ≥ 50% HTN = 20 (10F10M): 70 ± 4 years	Graded treadmill until exhaustion	SBP by sphygmomanometer	HFpEF > HTN at peak exercise
Namasivayam et al., 2022 (25)	HFpEF = 146 (72F74M): 63 ± 13 years, LVEF ≥ 50%, HFrEF = 57 (13F44M): 60 ± 13 years	Upright cycling until exhaustion	SBP and DBP by intra-arterial catheter	HFpEF > HFrEF at peak exercise
Borlaug et al., 2006 (15)	HFpEF = 17 (16F1M): 65 ± 9 years, LVEF ≥ 50%, $\dot{V}$O_2peak_ 9.0 ± 3.4 ml/kg/min CTRL = 19 (16F3M): 65 ± 9 years, $\dot{V}$O_2peak_ 14.4 ± 3.4 ml/kg/min	Upright cycling until exhaustion	SVRI was calculated from Gated 99mTc blood pool images	HFpEF < CTRL at peak exercise
Borlaug et al., 2010 (16)	HFpEF = 21 (16F5M): 67 ± 11 years, LVEF ≥ 50%, $\dot{V}$O_2peak_ 12.7 ± 3.1 ml/kg/min HTN = 19 (14F5M): 65 ± 11 years, history of BP >140/90 mmHg and treatment with ≥1 antihypertensive medication, $\dot{V}$O_2peak_ 13.8 ± 2.6 ml/kg/min CTRL = 10 (7F3M): 62 ± 7 years, no CVD and/or diabetes mellites, $\dot{V}$O_2peak_ 18.6 ± 3.3 ml/kg/min	Upright cycling until exhaustion	SVRI was calculated from BP by manual auscultation and cardiac output by transthoracic echo-Doppler.	HFpEF > CTRL and HTN at 20W exercise ΔSVRI were negatively correlated with $\dot{V}$O_2peak_
Sarma et al., 2021 (26)	HFpEF = 20 (12F8M): 69 ± 7 years, LVEF ≥ 50%, $\dot{V}$O_2peak_ 13.1 ± 3.4 ml/kg/min CTRL = 14 (7F7M): 72 ± 5 years, $\dot{V}$O_2peak_ 22.7 ± 4.0 ml/kg/min	Static handgrip until exhaustion, at 40% MVC	MBP by finger plethysmograph	HFpEF > CTRL at rest HFpEF = CTRL at peak exercise
Moriwaki et al., 2021 (27)	HFpEF = 10 (8F2M): 68 ± 18 years, LVEF 69 ± 9%, $\dot{V}$O_2peak_ 18 ± 4 ml/kg/min HFrEF = 10 (3F7M): 53 ± 11 years, LVEF 23 ± 6%, $\dot{V}$O_2peak_ 21 ± 7 ml/kg/min	Static handgrip for 3 min, at 30% MVC	MBP estimated by right heart catheter	HFpEF > HFrEF at rest HFpEF = HFrEF during handgrip
***Peripheral active muscle vasculature***				
Lee et al. 2016 (28)	HFpEF = 21 (10F11M): 68 ± 2 years, LVEF 61 ± 1% CTRL = 20 (10F10M): 71 ± 2 years	Single leg knee extension at 0, 5, 10 and 15 W for 3 min at each level	LBF and LVC at common femoral artery by ultrasound Doppler	HFpEF < CTRL at 10 and 15 W
Ratchford et al, 2020 (29)	HFpEF = 25 (14F11M): 69 ± 10 years, LVEF 63 ± 7% HTN = 25 (10F15M): 53 ± 7 years	Dynamic HG at 15, 30, and 45% MVC for 3 min each	FBF and FVC at brachial artery by ultrasound Doppler	HFpEF < HTN in FBF at 30 and 45% and in FVC at 45% MVC
Puntwangkoon et al., 2009 (14)	HFpEF and HFrEF = 12 (sex unclarity):70 ± 8 years (means ± SD), LVEF 48 ± 25%, $\dot{V}$O_2peak_ 13.9 ± 3.2 ml/kg/min CTRL = 13(sex unclarity): 67 ± 5 years, LVEF 66 ± 7%, $\dot{V}$O_2peak_ 19.8 ± 5.7 ml/kg/min	Incremental supine cycling exercise	Superficial femoral artery blood flow by magnetic resonance imaging	Both HFpEF and HFrEF < CTRL during exercise
Thompson et al. 2016 (30)	HFpEF = 5(sex unclarity): 67 ± 11 years, LVEF 56.6 ± 5.5%, $\dot{V}$O_2peak_ 18.5 ± 5 ml/kg/min HFrEF = 5(sex unclarity): 69 ± 9 years, LVEF 36.4 ± 12.0%, $\dot{V}$O_2peak_ 18.3 ± 2 ml/kg/min	Unilateral knee extension exercise at 85% of maximal weight for 4 min	Femoral venous blood flow by magnetic resonance imaging	HFpEF = HFrEF at the end of exercise

BF, burst frequency; BP, blood pressure; CTRL, control; CVD, cardiovascular disease; FBF, forearm blood flow; FVC, forearm vascular conductance; HF, heart failure; HFpEF, HF with preserved ejection fraction; HFrEF, HF with reduced ejection fraction; HTN, hypertension; LBF, leg blood flow; LVC, leg vascular conductance; LVEF, left ventricular ejection fraction; MAP, mean arterial pressure; MSNA, muscle sympathetic nerve activity; MVC, maximal voluntary contraction; NE, norepinephrine; PECA, post exercise circulatory arrest; SVRI, systemic vascular resistance index; ˙VO_2peak_, peak oxygen uptake.

## Sympathetic neural responses

Sympathetic outflow during exercise can be directly measured from peripheral nerves innervating the muscle vasculature as sympathetic neural discharge (muscle sympathetic nerve activity; MSNA) *via* microneurography (31, 32), or indirectly *via* plasma concentrations of norepinephrine (NE) (33–35).

A recent case report (22) assessed MSNA in one patient with HFpEF and one non-HF control (both 74 years, female) during dynamic single-leg cycling [0 W (mild) and 50% of $\dot{V}$O_2peak_ (moderate), 2 min/stages], finding that MSNA burst frequency (BF) increased (Δ) in the patient with HFpEF (ΔBF, mild: +13 and moderate: +16 bursts/min), yet was reduced in the non-HF control (mild: −6 and moderate: −7 bursts/min). Notably, previous study has demonstrated that patients with HFrEF also exhibit this paradoxical increase in MSNA BF [mild: +5 ± 4 and moderate: +7 ± 7 bursts/min ± standard deviation (SD)] during the same single-leg cycling protocol (36). These studies collectively indicate that the MSNA responses to dynamic exercise tend to be higher in patients with HFpEF compared to non-HF controls, and possibly patients with HFrEF, but studies comprising a larger cohort are needed to confirm this.

Contrary to the MSNA differences, patients with HFpEF exhibit similar increases in plasma NE concentrations to non-HF controls immediately after maximal-effort upright cycling, despite having a shorter exercise duration (15). Furthermore, Tsuchida et al. (24) compared the plasma NE responses to peak cycling in patients with preserved (≥45%) vs. reduced (<45%) LVEF, finding that patients with reduced LVEF had a lower peak exercise workload and duration than HF patients with preserved LVEF, yet no group differences in plasma NE concentrations were observed. Taken together, these two studies indicate that patients with HFpEF may exhibit greater sympathetic neurohormonal reactivity than non-HF controls, yet lower sympathetic outflow than patients with HFrEF for a given absolute workload. Importantly, plasma NE concentration provides a crude index of overall sympathetic activity. Circulating NE concentrations are dependent on both NE release from adrenergic nerve terminals and eventual removal from the circulation (33–35).

To our knowledge, only one assessment of the sympathetic responses to static handgrip exercise in one patient with HFpEF has been published. Bunsawat et al. (23) measured MSNA from the peroneal nerve in a patient with HFpEF (65 years, female) and a healthy individual (70 years, female) during static handgrip exercise at 30% and 40% of their maximal voluntary contraction (MVC) force for 2 min each. They reported that absolute MSNA BF in the patient with HFpEF tended to be higher at rest and throughout exercise, whereas the increases in MSNA BF (30% MVC: 7 vs. 10; 40% MVC: 22 vs. 24 bursts/min) seemed to be similar between the patient with HFpEF and the healthy control participant, respectively, during the last minute of exercise. Notably, though patients with HFrEF also exhibit greater absolute MSNA BF compared to controls (37), ΔMSNA during 30% MVC handgrip was higher in patients with HFrEF (ΔBF at 1-min: ∼5 ± 12, 2-min: ∼10 ± 12 bursts/min ± SD) than non-HF controls (ΔBF at 1-min: ∼1 ± 6, 2-min: ∼2 ± 4 bursts/min ± SD) (37). These responses in patients with HFrEF appears to be greater compared to that observed by Bunsawat et al. (23) in a patient with HFpEF (ΔBF at 1-min: 2, 2-min: 7 bursts/min). Taken together, these studies suggest that patients with HFpEF may exhibit smaller sympathetic neural reactivity to static exercise than patients with HFrEF, but similar reactivity to non-HF controls. Future studies with larger sample sizes are needed to confirm these preliminary observations.

In summary, patients with HFpEF and HFrEF exhibit greater sympathetic neural reactivity than non-HF controls during *dynamic* exercise, which may contribute to blunted increases in skeletal muscle blood flow and therefore exercise intolerance. Further, patients with HFpEF perhaps exhibit higher sympathetic responses to dynamic exercise than patients with HFrEF. Conversely, patients with HFpEF seem to exhibit smaller sympathetic responses than patients with HFrEF but similar responses to non-HF controls during *static* exercise. Sympathetic hyperactivity during exercise in patients with HFrEF is thought to be mainly attributed to exaggerated exercise pressor reflex activation (38, 39) including greater metabo- (37) and mechano-reflex activity (40) due to skeletal muscle abnormalities like muscle atrophy (41–43). Other compensatory responses include attenuated sympathoinhibition by cardiopulmonary (44) and arterial baroreceptors (45), heightened humoral activation (46), and enhanced chemoreflex activity (47). However, not all of these mechanisms may contribute to the heightened sympathetic activity in patients with HFpEF during exercise. The impact of the muscle metaboreflex in HFpEF remains equivocal with some studies demonstrating augmented (48) or unchanged (26) metaboreflex activation compared to non-HF controls, despite the observed muscle atrophy and biomolecular changes in patients with HFpEF (49). Moreover, Seravalle et al. (50) reported little to no baroreflex dysfunction in HFpEF, suggesting that this mechanism for sympathoexcitation in HFrEF may be different in HFpEF. Therefore, more work in larger samples is needed to confirm the augmented sympathoexcitation during *dynamic* exercise and the maintained sympathetic responses during *static* exercise in patients with HFpEF, and further clarify the underlying mechanisms.

## Hemodynamics

### Blood pressure

During dynamic exercise, where sympathetic neural responses are likely heightened, patients with HFpEF exhibit excessive pressor responses compared to non-HF controls and patients with HFrEF (8, 25). Kato et al. (8) found that the ΔBP during maximal treadmill exercise was greater in patients with HFpEF compared to individuals with hypertension, despite similar resting BP between groups and a shorter exercise duration in patients with HFpEF. Moreover, Namasivayam et al. (25) directly compared BP at peak exercise workload (upright cycle ergometer) between patients with HFrEF and HFpEF, finding that greater increases in systolic and diastolic BP in individuals with HFpEF. Furthermore, they found that pulsatile BP [the ratio of pulse pressure to systolic BP which indicates the power required to deliver blood to target organs (51)] was positively correlated with both arterial stiffness and SV in patients with HFpEF, but only SV in those with HFrEF. These studies indicate that the excessive BP response during dynamic exercise in patients with HFpEF is likely driven by heightened peripheral vascular dysfunction whereas the pressor response in patients with HFrEF is largely driven by cardiac function.

Previous studies have indicated that increases in peripheral vascular resistance predominantly account for excessive increases in exercise BP and exercise intolerance in patients with HFpEF. For instance, Borlaug et al. (15) reported that the decrease in systemic vascular resistance index during exercise in non-HF controls was attenuated in patients with HFpEF and the increases in cardiac index (i.e., $\dot{Q}$c scaled to body size) was also smaller compared to controls at low intensity (20 W), and peak exercise. The same group also reported (16) that the reduction in systemic vascular resistance (assessed from BP and $\dot{Q}$c during exercise was negatively corelated with $\dot{V}$O_2peak_ whereas positively correlated with cardiac function (i.e., HR and LV contractility). Altogether, these results indicate that abnormally high peripheral vascular resistance in patients with HFpEF, rather than cardiac dysfunction, may augment the BP response during exercise.

During static exercise, patients with HFpEF exhibit smaller BP responses compared to non-HF controls and HFrEF. Sarma et al. (26) measured BP during a 40% MVC static handgrip until exhaustion, finding that the peak BP responses to exercise were not different between patients with HFpEF and controls despite higher baseline BP in patients with HFpEF. This study indicates that ΔBP was smaller in patients with HFpEF than non-HF controls during exercise. Conversely, in patients with HFrEF, Notarius et al. (37) reported significantly higher ΔBP than non-HF controls in a 2-min bout of 30% MVC static handgrip exercise, despite similar resting BP between groups. Further, Moriwaki et al. (27) compared patients with HFpEF and HFrEF during 30% MVC static handgrip, finding that mean BP during exercise had no significant difference between groups despite patients with HFpEF exhibiting higher mean BP at rest, suggesting that the pressor responses to static exercise are smaller in patients with HFpEF than HFrEF. Together, unlike patients with HFrEF, individuals with HFpEF exhibit smaller BP responses during small muscle mass, static exercise compared to non-HF controls.

In summary, during *dynamic* exercise, smaller reductions in peripheral vascular resistance and/or impaired vasodilator capacity may predominantly contribute to excessive BP responses to exercise in patients with HFpEF, which may be due to excessive sympathetic neural responses (discussed later). Contrarily, during *static*, small muscle mass exercise, patients with HFpEF exhibit smaller BP responses than HFrEF and non-HF controls. However, thus far, it is unclear how these BP responses are linked to exercise intolerance in patients with HFpEF.

### Peripheral active muscle vasculature

To determine whether exercise intolerance in HF is peripherally determined, previous studies have assessed the peripheral vascular responses to single leg-knee extension or dynamic handgrip exercise as $\dot{Q}$c is not a limiting factor during small muscle mass exercise (52). For example, using doppler ultrasound to measure active leg blood flow (LBF) dynamics during single-leg extension exercise (0–5–10–15 W, 3 min/stage), Lee et al. (53) reported that despite similar BP responses in both groups, LBF and leg vascular conductance were lower in patients with HFpEF compared to non-HF controls, indicating that limb vasodilator function is impaired in this patient population. Similarly, findings were observed in the forearm vascular response comparing to hypertension controls during dynamic handgrip (15%–30%–45% MVC, 3mins/stage) (28). Also, patients with HFrEF exhibit lower LBF during the single-leg extension exercise protocol (0–5–10–15 W, 3 min/stage) (29). Puntawangkoon et al. (14) assessed LBF at the superficial femoral artery using magnetic resonance imaging (MRI) during submaximal supine cycling in patients with HFpEF and HFrEF as well as non-HF controls, finding smaller increases in LBF in both groups of HF compared to controls. Taken together, patients with HFpEF and HFrEF have impaired limb vascular function during exercise, which ultimately reduces oxygen delivery to active tissue and may relate to the exercise intolerance observed in HF.

Notably, when comparing the hemodynamic and LBF responses to single-leg extension exercise between patients with HFrEF (29) and HFpEF (53), the mean BP response appears to be relatively higher in HFpEF (15–20 mmHg) than in HFrEF (10–16 mmHg), but LBF was similar between groups, suggesting lower leg vascular conductance in patient with HFpEF vs. HFrEF. This lower conductance was corroborated by Thompson et al. (54) who directly compared femoral vein blood flow during submaximal single knee extension *via* MRI in a small number of subjects with HFpEF vs. HFrEF (*n* = 5, respectively). They reported no significant differences in venous LBF between patients with HFpEF and HFrEF. However, patients with HFpEF tended to have higher systolic (15 vs. 10 mmHg) and diastolic (12 vs. 9 mmHg) BP responses during exercise compared to patients with HFrEF. These studies suggest that patients with HFpEF require a higher arterial perfusion pressure to maintain a given LBF due to higher leg vascular resistance (i.e., lower leg vascular conductance) compared to patients with HFrEF, supporting the idea that peripheral factors may primarily account for exercise intolerance in patients with HFpEF.

Unlike dynamic exercise, to our best effort, we could not identify a study assessing blood flow during static exercise, likely related to the difficulty in assessing blood flow during sustained vascular compression in the active muscle.

## The relationship between peripheral vascular and sympathetic responses during exercise

Although no studies have directly assessed the relationship between peripheral vascular and sympathetic neural responses during exercise in patients with HFpEF, pharmacological studies have indicated that altered excessive sympathetically-mediated vasoconstriction primarily contributes to augmented BP responses during dynamic exercise, leading to exercise intolerance in patients with HFpEF. For instance, Kato et al. (8) treated patients with HFpEF and hypertension using candesartan, an angiotensin II receptor blocker (ARB), for 4 weeks and found that patients with HFpEF, but not hypertensive controls, had reduced peak systolic BP and longer time to exhaustion during maximal dynamic exercise. Further, following 1 year of perindopril [peripherally acting angiotensin converting enzyme (ACE) inhibitor] treatment, patients with HFpEF exhibited improvements in the 6-minute walk test (30). One conceivable mechanism relates to an ARB or ACE-inhibitor mediated suppression of excess sympathetic discharge, which could decrease peripheral vascular resistance and increase oxygen supply to the muscle (55) as well as functional capacity in patients with HFpEF. Although a previous systematic review and meta-analysis by Zheng et al. (12) did not demonstrate improvements in aerobic or functional capacity in patients with HFpEF following the use of ACE-inhibitors and ARBs, they did not include the two studies mentioned here. This highlights the issue that there are only a few studies examining therapies that may help mitigate the aberrant cardiovascular responses to exercise observed in patients with HFpEF. Nonetheless, these data provide some insight that sympathetic overexcitation coupled with exaggerated peripheral vasoconstriction during exercise may be one of the primary factors underlying exercise intolerance in patients with HFpEF.

The concept of functional sympatholysis is the ability of active skeletal muscle to override sympathetic vasoconstrictor drive, ensuring adequate oxygen delivery to the muscle (2, 3). While this physiological response is well-established in healthy humans, it is attenuated in diseases like elevated sympathetic activity (3). To our knowledge, only one study has assessed functional sympatholysis in patients with HF mildly reduced EF (45%), finding that the responsiveness to sympathetic vasoconstrictor (tyramine) during exercise was not altered while the peak vasodilatory capacity of ATP [a counter of sympathetic vasoconstriction elicited by exercise (56)] infusion was reduced compared to the non-HF controls (57). Whether sympatholysis is impaired in patients with HFpEF remains unknown and is an important area to clarify in the future

## Conclusion and future directions

The current data indicate that during *dynamic* exercise, patients with HFpEF exhibit higher peripheral vascular resistance, likely attributed to sympathetic hyperreactivity, than non-HF controls and perhaps patients with HFrEF, which may explain the larger increases in exercise BP and limited muscle blood flow. Conversely, during *static* exercise, patients with HFpEF exhibit smaller pressor responses than non-HF and HFrEF, despite the similar sympathetic responses compared to non-HF, suggesting that mechanisms other than sympathetic vasoconstriction may cause the lower workloads and shorter exercise durations observed in patients with HFpEF (see Table 2 for a summary of the conclusion). Altogether, this review highlights that our understanding about sympathetic neural control of the circulation and hemodynamic exercise in patients with HFpEF are still in its infancy and further work is needed to confirm the current understanding about sympathetic neural and hemodynamic responses to exercise in larger sample sizes. The research is also urgently required to clarify the pathophysiology lying between sympathetic neural activity and vasculature to develop treatment for exercise intolerance in this chronic disease that is growing in prevalence.

**Table 2:** Summary of sympathetic and hemodynamic responses to *dynamic* and *static* exercise in patients with HFpEF vs. HFrEF compared to non-HF controls.

	HFpEF	HFrEF
***Dynamic exercise***		
SNS activity	↑↑ (22)	↑ (36)
BP	↑↑ (8, 25)	↑ (25)
Muscle blood flow	↓ (28, 53)	↓ (29)
***Static exercise***		
SNS activity	↔ (23)	↑↑ (37)
BP	↓ (26)	↑ (37)
Muscle blood flow	?	?

SNS, sympathetic nervous system; BP, blood pressure; ↑, higher than controls; ↑↑, higher than both controls and another group; ↓, lower than controls; ↔, not different from controls; ?, not enough data.
